# Genomic signature to guide adjuvant chemotherapy treatment decisions for early breast cancer patients in France: a cost-effectiveness analysis

**DOI:** 10.3389/fonc.2023.1191943

**Published:** 2023-06-23

**Authors:** Elsa Curtit, Martine Marie Bellanger, Virginie Nerich, Delphine Hequet, Jean-Sebastien Frenel, Olivier Cristeau, Roman Rouzier

**Affiliations:** ^1^ University of Franche-Comté, University Hospital of Besançon J. Minjoz, INSERM, EFS UMR 1098, Besançon, France; ^2^ UMR CNRS6051, Ecole des Hautes Etudes en Santé Publique - School of Public Health (EHESP), University of Rennes, Rennes, France; ^3^ Department of Pharmacy, University Hospital of Besançon, France; INSERM, EFS-BFC, UMR 1098, University of Franche-Comté, Besançon, France; ^4^ Institut Bourdonnais, Clinique Saint Jean de Dieu, Paris, France; ^5^ Institut de Cancérologie de l’Ouest, Saint Herblain, France; ^6^ Creativ-Ceutical, Paris, France; ^7^ Centre François Baclesse, Caen, France

**Keywords:** genomic signatures, adjuvant chemotherapy, cost-effectiveness analysis, early breast cancer, decision impact

## Abstract

**Introduction:**

Chemotherapy (CT) is commonly used as an adjuvant treatment for women with early breast cancer (BC). However, not all patients benefit from CT, while all are exposed to its short- and long-term toxicity. The Oncotype DX^®^ test assesses cancer-related gene expression to estimate the risk of BC recurrence and predict the benefit of chemotherapy. The aim of this study was to estimate, from the French National Health Insurance (NHI) perspective, the cost-effectiveness of the Oncotype DX^®^ test compared to standard of care (SoC; involving clinicopathological risk assessment only) among women with early, hormone receptor-positive, human epidermal growth factor receptor 2-negative BC considered at high clinicopathological risk of recurrence.

**Methods:**

Clinical outcomes and costs were estimated over a lifetime horizon based on a two-component model that comprised a short-term decision tree representing the adjuvant treatment choice guided by the therapeutic decision support strategy (Oncotype DX^®^ test or SoC) and a Markov model to capture long-term outcomes.

**Results:**

In the base case, the Oncotype DX^®^ test reduced CT use by 55.2% and resulted in 0.337 incremental quality-adjusted life-years gained and cost savings of €3,412 per patient, compared with SoC. Being more effective and less costly than SoC, Oncotype DX^®^ testing was the dominant strategy.

**Discussion:**

Widespread implementation of Oncotype DX^®^ testing would improve patient care, provide equitable access to more personalized medicine, and bring cost savings to the health system.

## 1 Introduction

In France, breast cancer (BC) is the most prevalent cancer in women, with 58,083 new cases in 2020 (1). The most common subtype of BC, accounting for 70% of all female cases, is hormone receptor-positive (HR+), human epidermal growth factor receptor 2-negative (HER2−) BC (2, 3).

Surgery followed by adjuvant systemic therapy is the mainstay of treatment for early BC. International and French clinical guidelines recommend that, for patients with HR+, HER2−, early BC, adjuvant treatment includes endocrine therapy (ET) with or without chemotherapy (CT) (4–6); in real-world clinical practice in France, CT is used in approximately 44% of these patients (7). However, not all patients benefit from CT, while all are exposed to its short- and long-term adverse events (AEs) (8–10). Long-term AEs of CT in BC survivors are potentially serious and include an increased risk of chronic heart failure, coronary artery disease, and secondary cancers (11, 12). The AEs related to CT may negatively affect multiple aspects of patients’ lives, contributing to long-term quality of life (QoL) deterioration in BC survivors (13, 14).

The decision to include CT in the treatment regimen is based on clinicopathological criteria associated with BC prognosis. Meta-analyses failed to accurately identify the characteristics of patients who are likely to benefit from adjuvant CT (9). Gene expression signature (GS) quantifies the molecular expression of a panel of selected genes, usually within a tumor, to facilitate the identification of prognostic factors and the selection of the most appropriate treatment. The Oncotype DX Breast Recurrence Score^®^ test (Exact Sciences, Madison, WI, USA), hereafter referred to as the Oncotype DX^®^ test and the 21-gene signature, assesses the expression of 21 cancer-related genes in tumor tissue to estimate a Recurrence Score^®^ (RS) result ranging from 0 to 100, with higher scores indicating a greater risk of recurrence and better benefit of chemotherapy (15, 16)^1^.

The value of the Oncotype DX^®^ test was confirmed in two randomized controlled trials in women with HR+, HER2−, early BC: TAILORx, enrolling patients with node-negative (N0) disease, and RxPONDER, enrolling patients with 1–3 invaded lymph nodes (N1) (10, 17, 18). TAILORx demonstrated that women with HR+, HER2−, N0, early BC would not benefit from CT if 1) they have an RS ≤ 16 regardless of age or 2) have an RS ≤ 25 and are aged 50 or more (10, 17). RxPONDER demonstrated that, in women with N1 disease and RS result of 0–25, CT showed no benefits in postmenopausal women but improved both invasive disease-free and distant relapse-free survival in premenopausal women (18). Consequently, in N1 premenopausal patients, the benefit of chemotherapy across the RS range was sufficient to completely exclude these patients from the target population of the 21-gene signature. Based on the aforementioned clinical trial evidence, the use of the 21-gene signature is endorsed by both European and US clinical practice guidelines (5, 6, 19, 20).

Given its high prevalence, it is unsurprising that the economic burden of BC is substantial, having been estimated at €15 billion across the European Union and €2.5 billion in France alone in 2009 (21). Based on the recent trial results (10, 17, 18), the Oncotype DX^®^ test shows promise to alleviate the economic burden of BC through optimizing therapy; however, its up-to-date economic evaluation from the French perspective is lacking. Such an analysis could aid decision-makers in selecting the most optimal strategy, given the need to maximize health outcomes in a setting of limited healthcare resources and budget. The results of a cost-effectiveness analysis comparing two strategies are usually expressed as the difference in costs between the strategies per unit difference in clinical outcomes, called an incremental cost-effectiveness ratio (ICER). The quality-adjusted life year (QALY), which combines QoL and life expectancy, is usually used as a summary measure of clinical outcomes; therefore, the ICER is expressed as a cost per QALY gained. When the intervention is less costly and more effective than its comparator, it is the “dominant” strategy. When the intervention is more costly and less effective than its comparator, it is said to be “dominated” by its comparator. In both situations, the ICER is not computed. In a situation where the intervention is more costly and more effective than the comparator, or less costly and less effective, the ICER can be compared against the willingness-to-pay (WTP) threshold, representing the maximum amount that the local healthcare system can spend per unit health outcome (usually per QALY). The studied intervention is therefore considered cost-effective if the ICER is lower than the WTP and not cost-effective if the ICER is higher than the WTP.

The aim of this study was therefore to assess the cost-effectiveness of the Oncotype DX^®^ test compared to standard of care (SoC; involving clinicopathological risk assessment only) among patients in France with HR+, HER2−, early BC who were considered at high clinicopathological risk of distant recurrence.

## 2 Materials and methods

### 2.1 Overview of the analysis

We developed a model to assess the cost-effectiveness of the Oncotype DX^®^ test compared with SoC (clinicopathological risk assessment only) for therapeutic decision-making in women with HR+, HER2−, early BC and ≤3 positive nodes who were at high clinicopathological risk of distant recurrence.

The model comprised two components. Patients initially entered the model in the decision tree component, representing the adjuvant treatment choice (ET alone or ET+CT) guided according to the modeled therapeutic decision support strategy (Oncotype DX^®^ test or SoC). Patients exiting the decision tree entered a Markov state transition component representing the long-term BC patient pathway.

Three sub-populations were assessed: 1) premenopausal (<50 years old) women with N0 disease, 2) post-menopausal (≥50 years old) women with N0 disease, and 3) post-menopausal (≥50 years old) women with N1 disease. The sub-populations assessed excluded premenopausal women with N1 disease, in line with the results of the RxPONDER trial (18). For simplification, the age at menopause was assumed to be 50 years for all patients in the model.

The analysis was conducted from the French National Health Insurance (NHI) perspective and was developed according to the guidelines for economic evaluation from the Haute Autorité de Santé (HAS), the French health technology assessment body (22). The model therefore included only direct medical and non-medical costs, and all costs were valued from the perspective of the NHI.

Costs and clinical outcomes associated with the use of the 21-gene signature and SoC were estimated over a lifetime horizon in each sub-population of interest and subsequently aggregated across the sub-populations to assess the cost-effectiveness of Oncotype DX^®^ test compared to SoC in the entire target population.

### 2.2 Model structure

**Figure 1:**
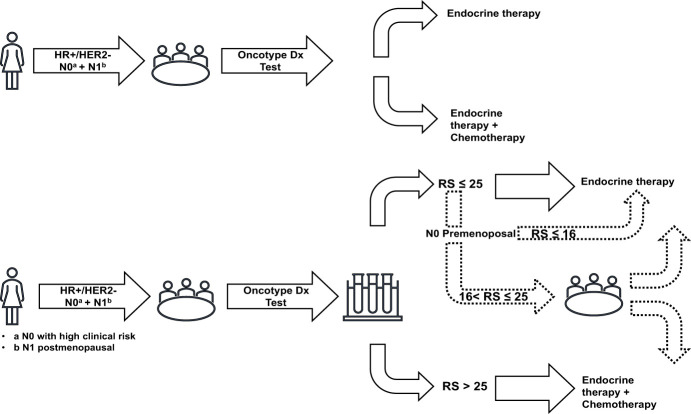
Decision trees representing the treatment choices in both therapeutic decision-making strategies. HER, human epidermal growth factor receptor; HR, hormone receptor; N0, node-negative disease; N1, 1–3 invaded lymph nodes; RS, recurrence score.

The initial decision tree component considered each sub-population and each therapeutic decision-making strategy separately to model the therapeutic choice of ET alone or ET+CT for each patient. The structure of the decision tree component is presented in **Figure 1**. With the Oncotype DX^®^ test strategy, patients were stratified according to their RS result, with each category having a specific probability of receiving CT:

* For premenopausal women with N0 disease, three RS categories were considered: <16, 16–25, and >25 (10).
* For postmenopausal women with N0 or N1 disease, two RS categories were considered: ≤25 and >25 (10, 18).

In the SoC strategy, the probability of receiving CT was dependent only on the sub-population (premenopausal women with N0 disease, postmenopausal women with N0 disease, or postmenopausal women with N1 disease) with no further stratification by RS result (10, 18).

**Figure 2:**
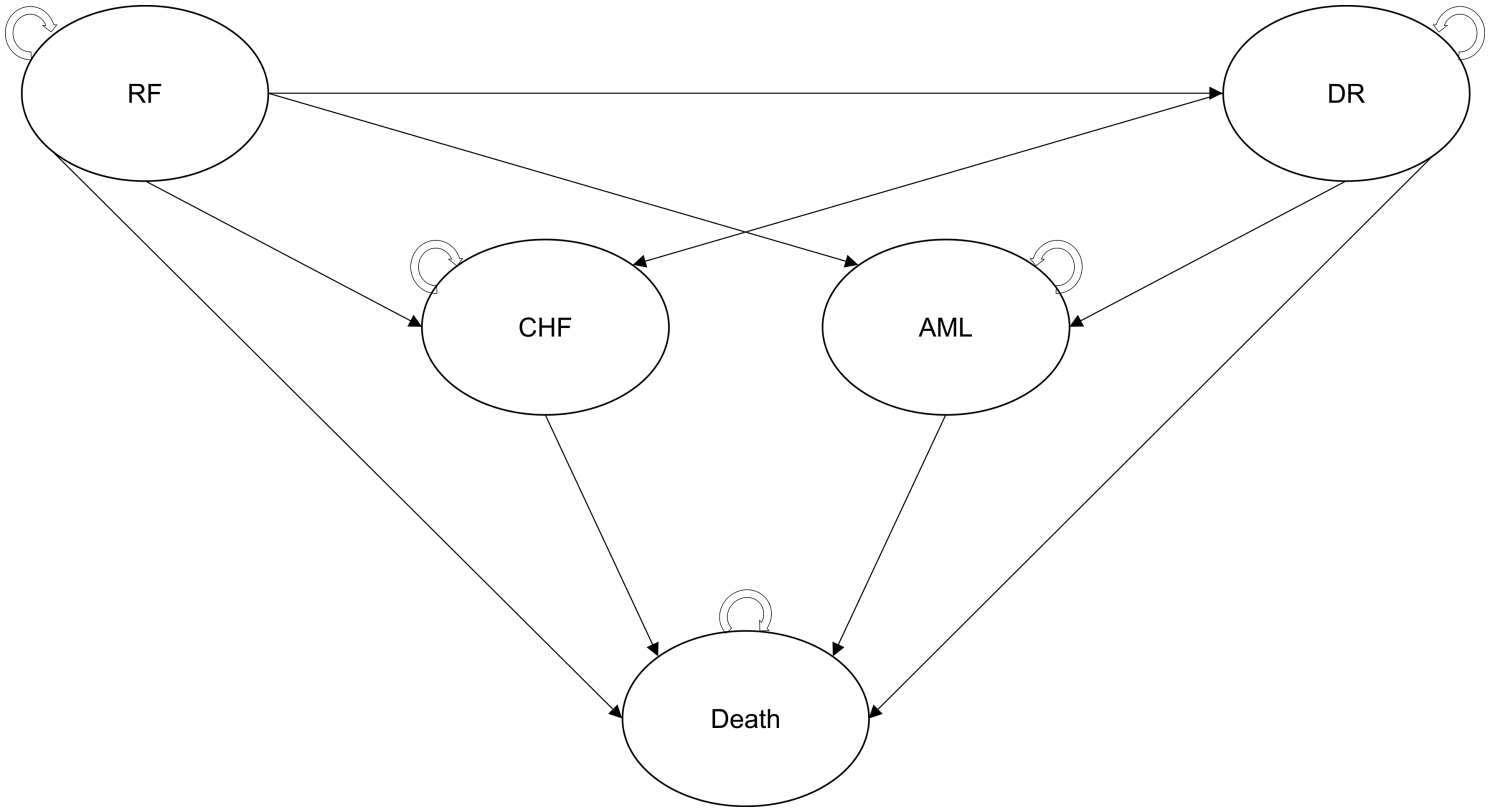
Structure of the Markov model. AML, acute myeloid leukemia; CHF, chronic heart failure, CT, chemotherapy; DR, distant recurrence; ET, endocrine therapy; RF, recurrence-free.

Patients exiting the decision tree component entered a Markov model utilizing a 6-month cycle length with half-cycle correction and including five health states (**Figure 2**):

* Recurrence-free: patients exiting the decision tree component initially entered this state and remained in it until their disease progressed; they experienced a severe long-term AE or died. The first year of this health state was modeled separately in order to consider higher follow-up costs and lower QoL related to CT.
* Distant recurrence: patients who experienced a metastatic recurrence of BC transitioned to this post-progression health state where they stayed until death or occurrence of a severe long-term AE. This health state was associated with higher costs and lower QoL than the recurrence-free state, reflecting the more severe condition. It was assumed that all patients in this state were treated with a cyclin-dependent kinase (CDK) 4/6 inhibitor.
* Acute myeloid leukemia (AML): despite being rare, AML is one of the most serious AEs that patients may develop following CT. Modeling it as a health state allowed us to consider increased mortality risk, acute and follow-up costs, and the associated reduction in QoL. Patients remained in this state until death.
* Chronic heart failure (CHF): patients who developed CHF after receiving CT transitioned to this health state, in which they remained until death. As for AML, this health state allowed us to consider the higher costs and worse QoL associated with the condition.
* Death was modeled as an absorbing health state. Costs related to end-of-life care were considered for all patients who died in the model.

### 2.3 Model outcomes

For each therapeutic decision-making strategy, the model estimated the outcomes for the three sub-populations of interest and computed the weighted average for the overall target population according to the distribution of patients in the sub-populations. Health economic outcomes were expressed per patient and included total and categorized costs (costs of Oncotype DX^®^ testing, adjuvant treatment, sick leave, transportation, end of life, and costs associated with each Markov health state), life-years (LYs), and QALYs. Incremental outcomes were expressed using standard metrics including:

* ICER, expressed as total costs per QALY (and LY) gained with the Oncotype DX^®^ test relative to SoC.
* Net monetary benefit (NMB), representing the total savings to the health system per patient undergoing Oncotype DX testing, estimated at a willingness-to-pay threshold of €20,000/QALY (23).

The following clinical outcomes were also assessed for each therapeutic decision-making strategy: the proportion of patients undergoing CT and the proportion of patients who developed AML and CHF. Costs and clinical outcomes were discounted at 2.5% per year for the first 30 years and at 1.5% thereafter, as recommended by HAS (22).

### 2.4 Population inputs

Patient distribution across sub-populations (**Table 1**) was derived from the French Early Breast Cancer Cohort (FRESH) and, for patients with N0 disease, adjusted for the proportion with high clinicopathological risk of recurrence reported in secondary analyses of the TAILORx trial (7, 17). In the 21-gene signature strategy, patients were further distributed across the RS categories based on the TAILORx trial for patients with N0 disease and the RxPONDER trial for patients with N1 disease (10, 17, 18).

**Table 1:** Population distribution and proportion of patients receiving CT by sub-population.

Sub-population	Percentage of the total target population	Oncotype DX^®^ test Distribution by RS (Percentage receiving CT by RS)			SoC Percentage receiving CT	Starting age
		<16	16–25 or ≤25^1^	>25		
N0 and age < 50 years^2^	41.2% (7, 17)	33.7% (0.0%) (10, 17)	73.9% (31.1%) (10, 17)	27.1% (100.0%) (10, 17)	60.8% (7, 24)	43 years (unpublished data)
N0 and age ≥ 50 years^2^	16.7% (7, 17)	Not applicable	71.8% (0.0%) (10, 17)	28.2% (100.0%) (10, 17)	29.4% (7, 24)	64 years (unpublished data)
N1 and age ≥ 50 years^2^	42.1% (7)	Not applicable	82.9% (0.0%) (18)	17.1% (100.0%) (18)	77.6% (7, 24)	61 years (unpublished data)

CT, chemotherapy; N0, node-negative disease; N1, 1–3 invaded lymph nodes; RS, recurrence score; SoC, standard of care.

^1^For premenopausal women, three RS categories were considered: <16, 16–25, and >25. For postmenopausal N0 and N1 sub-populations, only two RS categories were considered: ≤25 and >25.

^2^For simplification, menopause was assumed to occur at age 50.

The proportions of patients receiving CT in each sub-population (**Table 1)** for the SoC strategy were estimated from FRESH (7) and adjusted for increased CT use in patients at high clinicopathological risk of distant recurrent based on the odds ratio reported in PONDX, a real-world study of the 21-gene signature in France (24). For the 21-gene signature, the proportions of patients receiving CT in each sub-population (**Table 1**) were based on clinical expert recommendations that also took into account the benefits of CT observed in different RS groups in the TAILORx trial (17):

* All patients with RS >25 received CT.
* None of the patients aged ≥50 years with RS ≤25 received CT.
* Of N0 patients, 31.1% aged <50 years with RS 16–25 received CT.

### 2.5 Inputs related to BC recurrence

Probabilities of BC recurrence were independent of the therapeutic decision-making strategy; i.e., testing with the 21-gene signature did not impact treatment outcomes. BC recurrence probabilities were sourced from clinical trials assessing ET and ET+CT (**Table 2**):

* For patients with N0 disease, data from TAILORx were used for all RS categories (10, 17).
* RxPONDER provided the probabilities for patients with N1 disease and RS ≤25 (patients with RS > 25 were excluded from the trial after screening) (18).
* Data from the high-risk RS group in the TransATAC trial were used for patients with N1 disease and RS > 25 (25).

**Table 2:** Ten-year probability of remaining free from BC recurrence.

Population	Oncotype DX^®^ test						SoC	
	RS <16		RS 16–25 or ≤25^1^		RS >25			
	ET	ET+CT	ET	ET+CT	ET	ET+CT	ET	ET+CT
N0 and age < 50 years^2^	97.0% (10, 17)	97.8% (10, 17)	82.7% (10, 17)	91.7% (10, 17)	71.1% (10, 17)	83.3% (10, 17)	84.4% (10, 17)	91.5% (10, 17)
N0 and age ≥ 50 years^2^	Not applicable	Not applicable	90.4% (10, 17)	90.8% (10, 17)	70.2% (10, 17)	78.3% (10, 17)	84.7% (10, 17)	87.3% (10, 17)
N1 and age ≥ 50 years^2^	Not applicable	Not applicable	91.2% (18)	90.6% (18)	62.0% (25)	75.4% (25)	86.2% (10, 17)	88.0% (10, 17)

CT, chemotherapy; ET, endocrine therapy; N0, node-negative disease; N1, 1–3 invaded lymph nodes; RS, recurrence score; SoC, standard of care; BC, breast cancer.

^1^For premenopausal women, three RS categories were considered: <16, 16–25, and >25. For postmenopausal N0 and N1 sub-populations, only two RS categories were considered: ≤25 and >25.

^2^For simplification, menopause was assumed to occur at age 50.

Based on the findings of Pan et al., a constant rate of BC recurrence was applied in patients receiving ET (26). Following recommendations from clinical experts, a constant BC recurrence rate was also applied to patients receiving ET+CT.

### 2.6 Long-term toxicities of CT

The 6-month probability of developing AML following anthracycline-based CT was estimated by Moebus et al. (27). For the risk of developing CHF, no reliable age-dependent French data were identified. Therefore, for patients treated with ET only, UK age-specific incidences standardized to the European population were used (28). Following the methodology of Hall et al. (29), the increased risk of developing CHF in patients treated with anthracycline-based CT was accounted for by applying the risk ratio of cardiac mortality estimated by the Early Breast Cancer Trialists Collaborative Group (9). Key inputs related to the long-term toxicity of CT are presented in **Table 3**.

**Table 3:** Other key base case model inputs.

Parameter	Base case value
Model settings	
Time horizon^1^	Lifetime (57 years maximum)
Discount rate	
For costs (initial 30 years)	2.50% (22)
For costs (over 30 years)	1.50% (22)
For outcomes (initial 30 years)	2.50% (22)
For outcomes (over 30 years)	1.50% (22)
Long-term AEs of CT	
Probability of AML	0.62% (27)
HR for CHF with CT	1.61 (29)
Mortality	
Median OS in the DR state (months)	63.90 (30)
5-year AML death probability	76.00% (31)
Utilities	
Utility adjustment factor for France	1.043 (35)
Utility in	
Recurrence-free state	0.860 (62)
DR state	0.715 (62)
AML state	0.271 (63)
CHF state	0.551 (29)
Utility decrement for CT	0.040 (36)
Costs	
Test cost (Oncotype DX^®^ test)	€1,850 (46)
Recurrence-free cost – Year 1	
N0 and age <50 years	€3,777 (unpublished data)
N0 and age ≥50 years	€2,973 (unpublished data)
N1 and age ≥50 years	€3,444 (unpublished data)
Recurrence-free cost—Years 2–5	€604 (48)
Recurrence-free cost—Years 6+	€604 (48)
CDK 4/6 costs in the DR state	€14,395 (47)
Disease management cost in the DR state	€6,516 (48)
AML one-off cost	€23,970 (49)
AML subsequent cost	€5,806 (48)
CHF one-off cost	€1,888 (49)
CHF subsequent cost	€1,148 (48)
Terminal care	€4,606 (49)
Transportation costs—CT	
N0 and age < 50 years	€2,906 (unpublished data)
N0 and age ≥ 50 years	€1,738 (unpublished data)
N1 and age ≥ 50 years	€1,200 (unpublished data)
Transportation costs – ET	
N0 and age < 50 years	€255 (unpublished data)
N0 and age ≥ 50 years	€925 (unpublished data)
N1 and age ≥ 50 years	€337 (unpublished data)
Sick leave related to CT	
N0 and age < 50 years	€6,316 (unpublished data)
N0 and age ≥ 50 years	€5,235 (unpublished data)
N1 and age ≥ 50 years	€10,609 (unpublished data)
Additional costs due to CT^2^	
N0 and age < 50 years	€2,063 (unpublished data)
N0 and age ≥ 50 years	€4,479 (unpublished data)
N1 and age ≥ 50 years	€3,619 (unpublished data)
Costs of ET—N0 and age < 50 years	€91 (47)
Costs of ET—N0/N1 and age ≥ 50 years	€223 (47)

AML, acute myeloid leukemia; CDK, cyclin-dependent kinase; CHF, chronic heart failure; CT, chemotherapy; DR, distant recurrence; ET, endocrine therapy; HR, hazard ratio; N0, node-negative disease; N1, 1–3 invaded lymph nodes; OS, overall survival; RS, recurrence score; AEs, adverse events.

^1^Lifetime horizon was defined as sufficient for the patient cohort to reach 100 years of age. Therefore, the number of years in the model differed depending on the age at model entry and was 57 years for premenopausal women with N0 disease, 39 years for postmenopausal women with N1 disease, and 36 years for post-menopausal women with N0 disease.

^2^Additional costs due to CT were estimated from OPTISOINS01 data as the difference between the average cost per patient treated with CT and the average cost per patient not treated with CT. We assumed that they included all CT-related costs and associated adverse event costs.

### 2.7 Mortality

Mortality risk differed by model health state; key mortality inputs are listed in **Table 3**. Patients in the recurrence-free state were assumed to have the same risk of death as the general population. For patients in the distant recurrence state, the median survival observed in the MonaLEESA trial of the CDK 4/6 inhibitor ribociclib was used to estimate the probability of death (30).

Mortality for patients in the AML health state was sourced from Mounier et al. (31). Patients with CHF are also at higher mortality risk than the general population, especially in the first year following the event. This was considered in the model by applying a different excess mortality risk in the first year and subsequent years after the development of CHF, both based on Taylor et al. (32).

General population mortality rates for women in France were sourced from the National Institute of Statistics and Economic Studies (INSEE) (33)^2^ statistics.

### 2.8 QoL inputs

Baseline utilities at model entry (**Table 3**) were sourced from a recent UK-based study and adjusted to the French EQ-5D index population norms (34–37). A multiplier (ratio of utility at a given age to the baseline utility at model entry) was used to account for the age-related decrease in QoL in the modeled population.

Each health state was associated with a utility value representing the QoL of individuals in this state (**Table 3**). The adverse impact of CT on QoL was considered through a utility decrement of 0.040 based on Campbell et al. that was applied to all patients receiving CT (38).

### 2.9 Costs

All costs were valued in Euro 2022. Literature-derived historical costs were adjusted for inflation using the rates for healthcare products and services published by INSEE (39)^3^. Key cost inputs are presented in **Table 3**. Several cost inputs were sourced from an unpublished analysis of data from OPTISOINS01, a French multicenter, prospective, observational cohort study that collected resource use and cost data during the first year from diagnosis among 604 patients with early BC (40–47). The analysis was performed in a sub-population of 188 patients, corresponding to the population of interest, and included direct medical and non-medical costs (unpublished data). More details on this analysis are available in the **Supplementary Material.**

#### 2.9.1 Oncotype DX^®^ test and treatment costs

The price of the Oncotype DX^®^ test was €1,849.50 based on the 2022 French tariff (Référentiel des actes innovants hors nomenclature (RIHN), code N537) (48)^4^. The RIHN provides a permanent support system for innovative medical biology and anatomopathology. It allows for early and transitional management of innovative medical biology and anatomopathology procedures.

With regard to adjuvant treatment costs, the costs of CT were estimated from the OPTISOINS01 data and assumed to include both treatment and AE management costs. All patients treated with CT were assumed to receive it for 6 months; consequently, the costs of CT were applied only in the first model cycle. The costs of CT-related sick leave were also estimated from the OPTISOINS01 data and applied in the first model cycle only.

Costs of ET were not available from OPTISOINS01 and were estimated from unit drug prices retrieved from the French public database of medicines (BDM) (49)^5^. The dosing and frequency of administration were sourced from Summaries of Product Characteristics available in the BDM database (49), and the proportion of patients receiving ET was based on expert opinion. The average annual cost of CDK4/6 inhibitors was estimated analogously to the cost of ET.

#### 2.9.2 Health state costs

The first-year costs for patients from each sub-population in the recurrence-free state were sourced from the OPTISOINS01 data and included radiotherapy, drugs, imaging, consultations, hospitalizations (including for AEs), nursing, physiotherapy, psychological follow-up, and transportation. The costs for subsequent years were estimated from French 2020 open-source data compiled (Data-pathologies) for monitored women with BC, which included all medical and non-medical costs covered by the NHI (50)^6^.

The annual costs for patients in the distant recurrence health state were estimated from Data-pathologies using the average costs per patient with active BC (50).

The first-year costs of AML and CHF were assumed to correspond to the average cost of hospitalization for these events. Costs were estimated from the national tariff using the primary diagnosis ICD-10 codes C920 (acute myeloid leukemia) and I427 (cardiomyopathy due to drug and external agent) (51)^7^. For the following years in these health states, the model utilized the average cost per patient with “other active cancers” (€11,611/year) for AML and “chronic heart failure” (€2,296/year) for CHF from Data-pathologies (50).

#### 2.9.3 End-of-life costs

A one-off end-of-life cost of €4,606 was applied to all patients in the model who died. This was estimated from the T2A 2022 tariff using the ICD-10 code Z515 (encounter for palliative care) as the primary diagnosis (51).

### 2.10 Uncertainty

Deterministic and probabilistic sensitivity analyses (DSA and PSA, respectively) were conducted. In DSA, each parameter was varied individually, using the low and high ranges of plausible values available in **Supplementary Table 2**, to assess the impact of specific parameters on model results and identify the most influential parameters. The results are presented as tornado diagrams displaying separately the 10 parameters that were most influential on cost and QALY estimates.

PSA was conducted to assess the overall level of uncertainty around model outcomes. All parameters were simultaneously sampled from plausible distributions detailed in **Supplementary Table 2** and the model run for 5,000 simulations. Incremental costs and QALYs were estimated for each simulation and averaged to compute the probabilistic ICER. The results of the PSA are presented as a cost-effectiveness acceptability curve, showing the probability of the 21-gene signature being cost-effective at different willingness-to-pay thresholds, and as a cost-effectiveness plane, displaying the spread of the ICERs obtained in individual PSA simulations.

In addition to the sensitivity analyses, two scenario analyses were conducted:

* Scenario 1: data from the Clalit registry were used to compute the distribution of RS and probabilities of receiving CT in the Oncotype DX^®^ strategy (52, 53).
* Scenario 2: in the Oncotype DX^®^ test strategy, alternative probabilities of receiving CT were used, based on expert opinion representing current clinical practice in France.

## 3 Results

### 3.1 Base case results

Average discounted costs per patient accumulated over the lifetime horizon are presented for each strategy in **Table 4**. Total costs were lower for the 21-gene signature than SoC (€66,554 and €69,966), resulting in incremental savings of €3,412 per patient. The additional cost of the test was offset by lower costs associated with CT-related sick leave (savings of €3,274), adjuvant treatments (savings of €1,106), and severe AEs (savings of €792).

**Table 4:** Average discounted costs per patient in the target population.

Costs	Oncotype DX^®^ test	SoC	Incremental
Test	€1,850	€0	€1,850
Adjuvant treatments^1^	€2,454	€3,559	−€1,106
Transportation	€931	€1,388	−€457
Sick leave due to CT	€2,032	€5,306	−€3,274
Recurrence-free	€26,160	€25,532	€628
Distant recurrence	€28,413	€28,705	−€292
AML	€453	€1,007	−€553
CHF	€1,827	€2,065	−€239
End of life	€2,434	€2,404	€31
**Total**	**€66,554**	**€69,966**	**−€3,412**

AML, acute myeloid leukemia; CHF, chronic heart failure; CT, chemotherapy; SoC, standard of care.

^1^ ET only or ET+CT.

Clinical and cost-effectiveness outcomes in specific populations are presented in **Table 5**. Testing with the 21-gene signature improved health outcomes when compared to SoC, with 14.84 *vs.* 14.50 QALYs and 18.96 *vs.* 18.59 LYs accrued per patient over a lifetime horizon for the two therapeutic decision-making strategies. Therefore, the 21-gene signature resulted in 0.337 incremental QALYs and 0.376 LY gained per patient (**Table 5**). The use of the 21-gene signature reduced CT use by 55.2% and, consequently, decreased the occurrence of AML and CHF by 55.1% and 11.9%, respectively. Additionally, patients undergoing Oncotype DX^®^ testing spent approximately 6 months more in the recurrence-free state than patients in the SoC arm of the model.

**Table 5:** Clinical and cost-effectiveness outcomes for the Oncotype DX^®^ test *vs.* SoC in the three sub-populations considered in the model.

Population	ΔCosts	ΔQALYs	ICER €/QALY	NMB	CT avoided	AML avoided	CHF avoided
Overall population	−€3,412	0.337	Dominant	€10,151	55.2%	55.1%	11.9%
N0 and age <50 years	€780	0.338	€2,309	€5,979	35.4%	40.2%	9.0%
N0 and age ≥50 years	−€734	0.134	Dominant	€3,406	4.1%	9.1%	0%
N1 and age ≥50 years	−€8,586	0.417	Dominant	€16,917	78.0%	79.8%	18.2%

AML, acute myeloid leukemia; CHF, chronic heart failure; ICER, incremental cost-effectiveness ratio; N0, node-negative disease; N1, 1–3 invaded lymph nodes; NMB, net monetary benefit; QALY, quality-adjusted life year; SoC, standard of care.

Being more effective and cost-saving compared to SoC, the Oncotype DX^®^ test was the dominant strategy. Assuming a willingness to pay 20,000€/QALY, the NMB of the 21-gene signature was estimated at €10,151 per patient.

In premenopausal women with N0 disease, the 21-gene signature was more expensive (additional cost of €780) but also more effective (0.338 QALYs gained) compared with SoC. The ICER in this sub-population was estimated at €2,309 per QALY gained; therefore, the 21-gene signature could be considered cost-effective at the €20,000/QALY threshold. In the remaining two sub-populations, the Oncotype DX^®^ test was the dominant strategy. In post-menopausal women with N0 disease, the Oncotype DX^®^ test was cost-saving (savings of €734) and associated with a gain of 0.134 incremental QALYs when compared to SoC. In postmenopausal women with N1 disease, the use of the 21-gene signature resulted in a gain of 0.417 QALYs and savings of €8,586. Across the three sub-populations, the reduction in CT use ranged from 4.1% in postmenopausal women with N0 disease to 78.0% in postmenopausal women with N1 disease; the reductions in AML and CHF occurrence followed a similar pattern (**Table 5**).

### 3.2 Scenario analyses

In scenario 1, using the real-world data from the Clalit registry (52, 53), the Oncotype DX^®^ assay remained the dominant strategy, associated with a gain of 0.192 QALYs and savings of €1,686 when compared to SoC. However, both the QALY gain and the cost savings were slightly lower than in the base case analysis.

In scenario 2, in which the probabilities of receiving CT were informed by clinical expert opinion representing current French clinical practice, the 21-gene signature was again dominant, resulting in cost-savings of €2,853 and a gain of 0.298 QALYs relative to SoC. The QALY gains and cost savings in this scenario were also somewhat smaller than in the base case analysis.

### 3.3 Sensitivity analyses

The model parameters that had the greatest impact on incremental costs in DSA were the costs related to CT, including sick leave, transportation, and treatment-related costs (**Supplementary Figure 1**). Parameters with the greatest impact on incremental QALYs were the time horizon, starting age of patients, discount rate for health outcomes, and probability of developing AML (**Supplementary Figure 2**). None of the parameters, when varied across their plausible ranges, changed the direction of model results; i.e., the Oncotype DX^®^ test remained cost-effective in all analyses performed.

**Figure 3:**
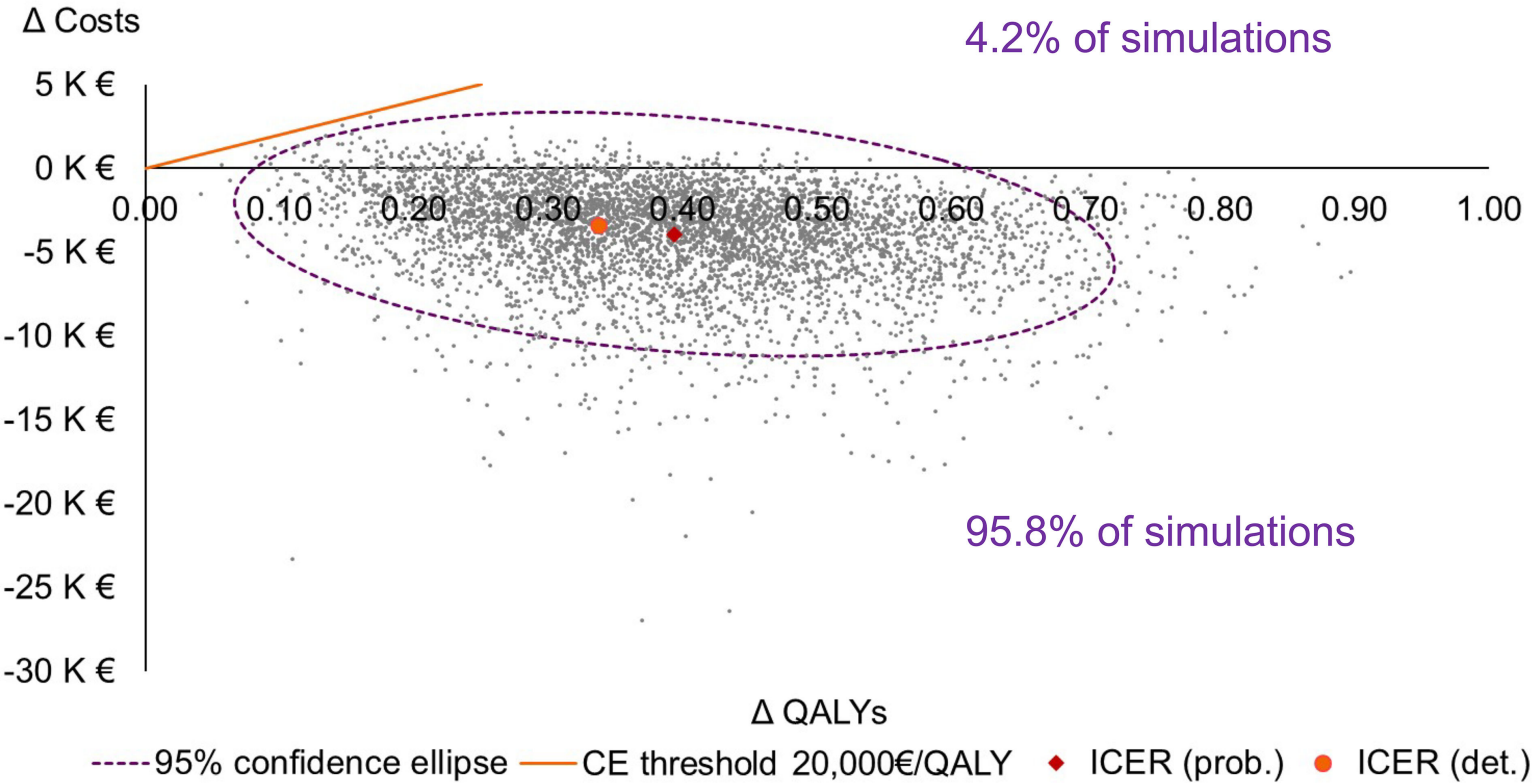
Cost-effectiveness plane. Note that only the northeast and southeast quadrants of the plane are presented. CE, cost-effectiveness; det., deterministic; ICER, incremental cost-effectiveness ratio; K, thousand; prob., probabilistic; QALY, quality-adjusted life-year.

In 95.8% of the PSA simulations, the Oncotype DX^®^ test was dominant (more effective and less costly) when compared to SoC (**Figure 3**). In the remaining 4.2% of simulations, the 21-gene signature was cost-effective when compared to SoC at a willingness-to-pay threshold of €20,000/QALY, being more costly and more effective than SoC (**Figure 3**). Across all PSA simulations, the average cost savings associated with the 21-gene signature were €3,926, and the average QALY gain was 0.394; therefore, the Oncotype DX^®^ test was the dominant strategy compared with SoC.

**Figure 4:**
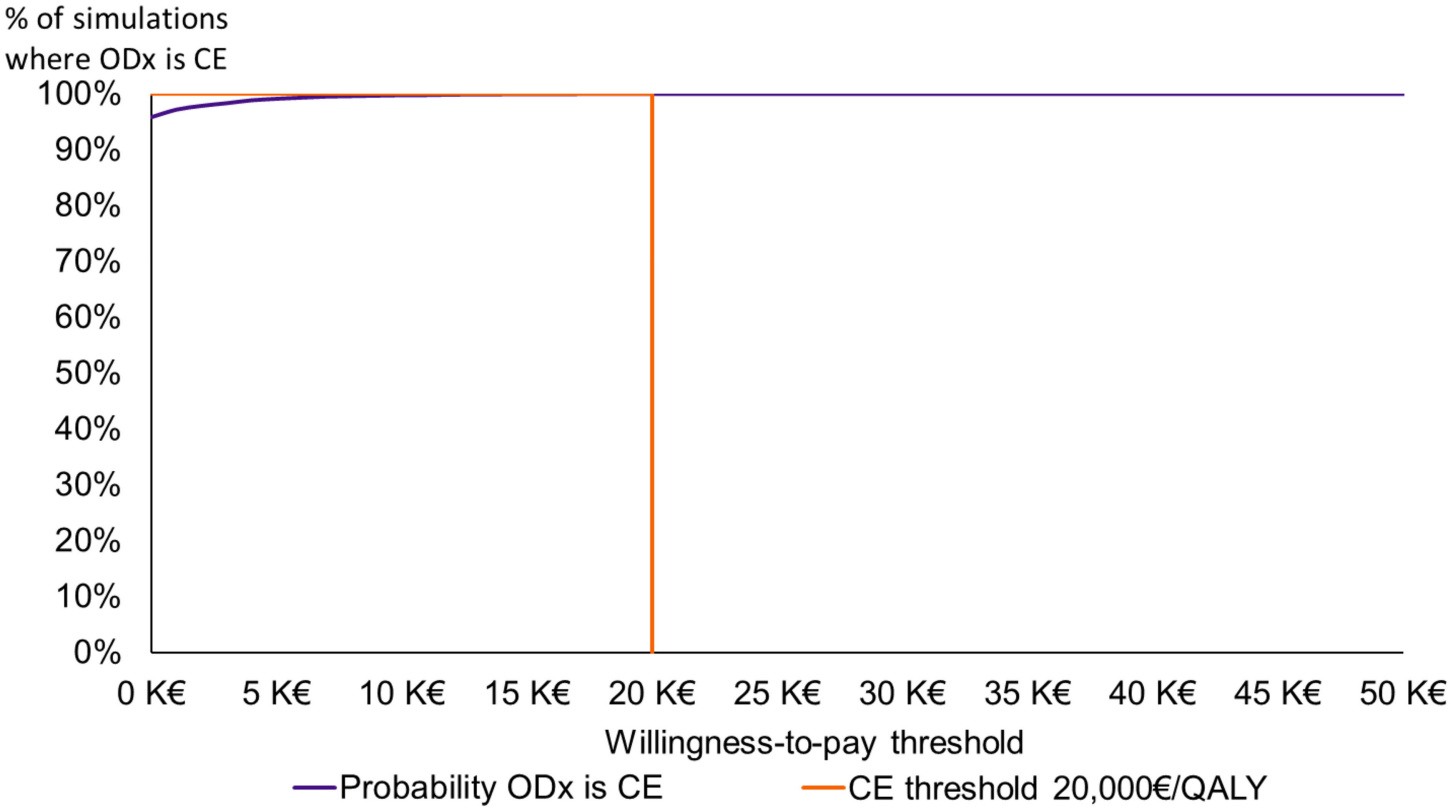
Cost-effectiveness acceptability curve. CE, cost-effective; K, thousand; ODX, the Oncotype DX^®^ test; QALY, quality-adjusted life-year.

On the cost-effectiveness acceptability curve, the probability of the Oncotype DX^®^ test being cost-effective against SoC was 99% at a willingness-to-pay threshold of €5,000/QALY and 100% at a threshold of €20,000/QALY (**Figure 4**).

## 4 Discussion

### 4.1 Summary of results

The use of the Oncotype DX^®^ test for therapeutic decision-making in patients with HR+, HER2−, early BC proved to be dominant (i.e., associated with improved clinical outcomes and lower costs) compared with SoC involving therapeutic decisions being made solely based on clinicopathological characteristics. Importantly, the use of the 21-gene signature reduced the use of CT by over half, with a corresponding reduction in the long-term AEs of CT. Despite the reduction in CT use with the 21-gene signature, clinical outcomes associated with this strategy were more favorable compared with SoC, strongly suggesting that the use of the 21-gene signature optimally targeted the use of CT only in those patients who can benefit from it, while avoiding unnecessary CT use. The benefit of the 21-gene signature was evident in all sub-populations assessed in the model, i.e., both pre- and postmenopausal patients with N0 HR+ HER2− disease and postmenopausal patients with N1 HR+ HER2− disease. Rigorous sensitivity analyses demonstrated the robustness of model results.

It is interesting to note that, despite a low percentage of CT avoided (4.1%) in the sub-population of postmenopausal patients with N0 disease, the Oncotype DX^®^ test was still found dominant against SoC. In FRESH, only 29% of patients in this sub-population received CT (7), suggesting that clinicians hesitate to recommend CT due to the low survival benefit in relation to patient age and the substantial burden of treatment. It is therefore this sub-population particularly in which the 21-gene signature could substantially facilitate targeting CT only to those patients who are likely to benefit from the treatment. The result on the sub-population of postmenopausal patients with N0 disease could reflect the situation where GS would be used on all comers, reducing the number of CT avoided compared to the situation where the Oncotype DX^®^ test is prescribed to a targeted population.

### 4.2 Comparison to published evidence

In a 2019 report, HAS evaluated the clinical utility of GS in patients with HR+, HER2−, early BC in France and concluded that, due to limited effectiveness data available, there was no direct evidence for the clinical utility of the Oncotype DX^®^ test when added to SoC (54). However, the analysis conducted by HAS was based on the evidence available before 2019, when the RxPONDER trial was still in progress. In comparison, our analysis was based on more recent data, including the RxPONDER trial (18), and real-world evidence from large studies representative of French clinical practice, such as PONDx (24) and FRESH (7). The conclusion from our analysis was very different than that reached by HAS. In the base case and all sensitivity and scenario analyses, the 21-gene signature was more effective than SoC. It was also the less costly strategy in both the base case analysis and the majority of PSA simulations and, therefore, dominant over SoC in terms of cost-effectiveness.

Two older studies based on early data also assessed the cost-effectiveness of the Oncotype DX^®^ test from the French healthcare payer perspective in women with HR+, HER2−, N0, early-stage BC, a sub-population of our analysis. Vataire et al. found the Oncotype DX^®^ test to be dominant over SoC, resulting in both lower costs (savings of €570 per patient) and higher effectiveness (0.14 QALYs gained per patient) (55). In a subsequent update of this study published by Katz et al., the 21-gene signature was associated with an additional cost of €352 and a gain of 0.17 QALYs, resulting in an ICER of €2,134/QALY; therefore, the Oncotype DX^®^ test was highly cost-effective in this analysis (56). It should be noted that the models developed by Vataire et al. and Katz et al. used a higher cost of the 21-gene signature (€3,180) than those used in our analysis (€1,849.50) (55, 56). The results of our analysis further confirm the findings of Vataire et al. and Katz et al., also demonstrating the cost-effectiveness of the Oncotype DX^®^ test relative to SoC.

Two additional cost-effectiveness evaluations of the Oncotype DX^®^ test have been performed from the perspective of the National Health Service (NHS) and Personal Social Services (PSS) in the UK. The recent evaluation by Berdunov et al. compared the Oncotype DX^®^ test to clinical risk tools in both pre- and postmenopausal women with HR+, HER2−, N1, early BC (34). In this analysis, the 21-gene signature was associated with a substantial reduction in CT usage and is more effective and less costly when compared to SoC (34); therefore, the results were similar to those observed in postmenopausal women with HR+, HER2−, N1, early BC in our analysis. However, it should be noted that the analysis by Berdunov et al. included both pre- and post-menopausal women with N1 disease (34), while our analysis focused on the post-menopausal population only, as the RxPONDER trial demonstrated that the benefits of CT in premenopausal women with N1 disease are sufficient to exclude these patients from Oncotype DX^®^ testing (18).

In 2018, the use of the Oncotype DX^®^ test in patients with HR+, HER2−, N0 or N1, early BC was also assessed by the National Institute for Health and Care Excellence (NICE) (57). In the base case analysis conducted as part of the NICE appraisal, the 21-gene signature was assumed to be a solely prognostic tool with no ability to predict the benefit of CT (57). The impact of including a predictive benefit of the Oncotype DX^®^ test was, however, assessed in sensitivity analyses (57). In the model developed as part of the NICE appraisal, the ICER relative to SoC (risk assessment based on clinicopathological features) was equal to £122,725/QALY gained in patients with N0 disease and a Nottingham Prognostics Index (NPI) of ≤3.4 when the predictive benefit of the 21-gene signature was excluded (57). In the scenario analysis including the predictive benefit of the 21-gene signature, the ICER in this population reduced to £34,245/QALY (57). In patients with N0 disease and NPI > 3.4 or N1 disease, the Oncotype DX^®^ test was dominated by SoC when the predictive benefit of the test was excluded but, conversely, dominated SoC in a sensitivity analysis including the predictive benefit of the 21-gene signature (57). Therefore, the results of the NICE model were very strongly dependent on whether the predictive benefit of the 21-gene signature was considered. Our model was consistent with the structural choices made in the NICE model (57); however, it utilized more recent data sources. Most importantly, in our study, the predictive benefit of the Oncotype DX^®^ test was included in the base case analysis, in which the Oncotype DX^®^ test was dominant or highly cost-effective compared with SoC. The inclusion of the predictive benefit of the 21-gene signature in our base case analysis is well supported by recent evidence from large and rigorously conducted TAILORx and RxPONDER trials (10, 17, 18).

Additionally, our findings were consistent with data published for countries outside Europe, i.e., Canada, Mexico, and Brazil. In Canada, Hannouf et al. in 2012 (58) and Hannouf et al. in 2014 (59) showed that the 21-gene signature would be cost-effective when compared to SoC: dominant, ICER of CAD 60,000 per QALY gained, and ICER of CAD 464 per QALY gained for N0 premenopausal, N0 postmenopausal, and N1 women. Bargalló-Rocha et al. in 2015 (60) demonstrated that the 21-gene signature was cost-effective when compared to SoC in the Mexican healthcare system for our population of interest: MXN 25,244 per LY gained. In Brazil, the publication of Mattar et al. (61) demonstrated that the 21-gene signature reduced CT use by 63%, which aligned with our findings.

### 4.3 Strengths

The strengths of our analysis include the use of the most recent data available both from the clinical trial (TAILORx (10, 17) and RxPONDER (18)) and real-world (PONDx (24), Data-pathologies (50), OPTISOINS01 (40), and FRESH (7)) settings. The most severe long-term toxicities of CT were modeled as health states, which allowed us to consider the costs, QoL losses, and increased mortality associated with these conditions. When interpreting the data, it should be noted that the perspective of our analysis was that of the French NHI, the model excluded indirect costs and outcomes, such as reduced QoL of patients’ family members and caregivers, productivity losses, and informal and formal caregiver costs. Taking into account the societal perspective would likely result in a larger estimated saving of the Oncotype DX^®^ test.

### 4.4 Limitations

To capture the patient pathway in a complex disease such as BC, several assumptions had to be made in the model. Initially, we sought to obtain the menopausal status of patients from FRESH (7); however, this was not reported. Therefore, the age at menopause in the model was arbitrarily set at 50 years, an assumption validated by clinical experts. Literature-derived model inputs were, in some cases, obtained from populations slightly differing from those of our model. Data from the high-risk RS group in the TransATAC trial were used to estimate the probability of BC recurrence in patients with N1 disease and RS > 25; however, it should be noted that high-risk patients in TransATAC were classified as those with an RS > 30 (25). The long-term costs for patients who developed AML were assumed to be equal to the average cost per patient with “other active cancers” from Data-pathologies (50) since AML-specific costs were not identified. Furthermore, the data on CT use from FRESH were not specific to patients at high clinicopathological risk of recurrence and required adjustment based on PONDx data (7, 24). The costs in the first modeled year were estimated from the French real-world OPTISOINS01 study (unpublished data). While the study included 604 women with BC, only 188 patients corresponded to the modeled population, and this relatively low number of patients meant that the costs obtained in the analysis of OPTISOINS01 data were highly variable. Nevertheless, the effect of this variability was assessed in the sensitivity analyses and had a very limited impact on the results of the model. It should also be noted that the data from FRESH (7), a source of several model inputs, may include patients for whom the treatment decision was based on the result of a GS test. The use of FRESH data can therefore be considered conservative, as it may introduce bias against the 21-gene signature, decreasing the usage of CT in the SoC strategy and therefore potentially lowering the estimated proportion of CT avoided in our analysis. It should be noted that despite the assumptions made during the development of the model and the uncertainty associated with some of the inputs, sensitivity and scenario analyses demonstrated that the model results were robust to changes in input parameters, with the Oncotype DX^®^ test proving dominant or cost-effective in all analyses conducted.

## 5 Conclusions

The results of this study demonstrated that routine use of the Oncotype DX^®^ test in women with HR+, HER2−, N0 or N1, early BC at high clinicopathological risk of distant recurrence would be associated with a substantial reduction in CT use, improved QoL, and lower costs when compared to SoC comprising clinicopathological risk appraisal only. In line with the intended use of the 21-gene signature, the model did not assume an effect of the test on treatment outcomes but rather focused on the ability of the 21-gene signature to predict the benefit of CT and thus tailor the use of CT to those patients who would benefit from it the most. The use of the 21-gene signature can therefore optimize care for women with BC, allowing patients to avoid unnecessary chemotherapy and its potentially life-threatening toxicities without impairing survival. Widespread implementation of the Oncotype DX^®^ test testing in women in France with HR+, HER2−, N0 or N1, early BC would improve patient care, provide equitable access to more personalized medicine, and bring cost savings to the health system. Future real-world research should aim to confirm the estimates from our model and address remaining evidence gaps. Furthermore, additional clinical research and associated health-economic evaluations on specific subgroups (e.g., younger women and racial/ethnic groups) could be of interest. Although differences in risks across racial groups were recently identified—Albain et al. (2021) (62) on TAILORx and more recently the 2022 San Antonio Breast Cancer Symposium presentation (63) on RxPONDER—findings need to be confirmed, and more robust data will be needed to conduct related cost-effectiveness studies.

## Data availability statement

The original contributions presented in the study are included in the article/**Supplementary Material**. Further inquiries can be directed to the corresponding author.

## Author contributions

All authors contributed to the conceptualization of the study, selection of the most appropriate methodology, formal analyses, model validation, and the preparation, review, and editing of the manuscript. DH and RR sourced the OPTISOINS01 data. All authors contributed to the article and approved the submitted version.

## Supplementary material

Supplementary Information Estimation of first year of “Recurrence free” and CT-related costs;

Supplementary Table 1 Average number of CT cures in each population.

Supplementary Table 2 Model parameters with DSA ranges and PSA distributions

Supplementary Figure 1 DSA results presenting the 10 model parameters that were most influential on the costs estimates

Supplementary Figure 2 DSA results presenting the 10 model parameters that were most influential on the QALY estimates.

1
https://precisiononcology.exactsciences.com/healthcare-providers/treatment-determination/breast-cancer/oncotype-dx-breast-recurrence-score/interpret-the-results

2
https://www.insee.fr/fr/statistiques/3311422?sommaire=3311425

3
https://www.insee.fr/fr/statistiques/serie/001763845#

4
https://solidarites-sante.gouv.fr/systeme-de-sante-et-medico-social/recherche-et-innovation/rihn

5
http://www.codage.ext.cnamts.fr/codif/bdm_it/

6
https://data.ameli.fr/pages/data-pathologies/

7
https://www.atih.sante.fr/tarifs-mco-et-had
