# Supplementary material for: Genomic signature to guide adjuvant chemotherapy treatment decisions for early breast cancer patients in France: a cost-effectiveness analysis
Source: Front Oncol. 2023 Jun 23;13:1191943. doi: 10.3389/fonc.2023.1191943 (PMC10327821; doi:10.3389/fonc.2023.1191943)
Supplement: Supplementary Information — Estimation of first year of “Recurrence free” and CT-related costs; [file DataSheet_1.docx]

Supplementary Material

# Estimation of first year of “Recurrence free” and CT-related costs

Costs of the first year of the Recurrence free health state and costs related to CT were estimated from OPTISOINS01, a French multicenter, prospective, observational cohort study aiming to identify the main care pathway of early BC from diagnosis to 1-year follow-up, and to evaluate costs from different perspectives (hospital and health insurance) (1-8, Unpublished data).

The study was divided in 4 phases: preoperative, surgery, adjuvant treatment and follow-up. The resources used in each phase were collected, including, among others, consultations, drugs, imaging, biopsy-puncture, extension assessment, pre-therapeutic assessment, radiotherapy, hospitalizations, supportive care (nursing, physiotherapy, psychological follow-up, etc.), as well as type of CT and number of CT treatment cycles (Supplementary Table 1). Additionally, sick leaves and transportations were recorder. Based on these data, a micro-costing analysis was conducted to estimate the costs in each phase for each patient.

Cost of CT treatments was estimated for each patient considering the frequency of administration (Supplementary Table 1), and the GHS 9606 for “Chemotherapy for tumor, in sessions” (GHM 28Z07Z), which was associated with a cost of 406.61€ in 2016 (9).

For this study, we had access to various patient-level aggregated costs, such as direct medical costs, direct non-medical costs, transportation costs, and sick leave costs for each phase. The patients entering our model were assumed to be post-surgery, therefore only the data for the phases 3 and 4 of OPTISOINS01 were used in this study.

For the model, the cost of the first year following surgery, i.e., first year in the Recurrence free health state, was estimated as the average of the sum of direct medical and non-medical costs of phases 3 and 4 for patients who received ET only. For patient receiving CT, the additional cost due to CT was estimated as the difference between the average cost per patient treated with CT and the average cost per patient not treated with CT; this cost was assumed to include treatments, monitoring and AEs costs.

References

1. Héquet D, Huchon C, Soilly A-L, Asselain B, Berseneff H, Trichot C, et al. Direct medical and non-medical costs of a one-year care pathway for early operable breast cancer: Results of a French multicenter prospective study. PLOS ONE. 2019;14(7):e0210917. doi: 10.1371/journal.pone.0210917.

2. Baffert S, Hoang HL, Bredart A, Asselain B, Alran S, Berseneff H, et al. The patient-breast cancer care pathway: how could it be optimized? BMC Cancer. 2015;15:394. doi: 10.1186/s12885-015-1417-4.

3. Hequet D, Huchon C, Baffert S, Alran S, Reyal F, Nguyen T, et al. Preoperative clinical pathway of breast cancer patients: determinants of compliance with EUSOMA quality indicators. Br J Cancer. 2017;116(11):1394-401. doi: 10.1038/bjc.2017.114.

4. Cariou A, Rouzier R, Baffert S, Soilly AL, Hequet D. Multidimensional impact of breast cancer screening: Results of the multicenter prospective optisoins01 study. PLoS One. 2018;13(8):e0202385. doi: 10.1371/journal.pone.0202385.

5. Arfi A, Baffert S, Soilly AL, Huchon C, Reyal F, Asselain B, et al. Determinants of return at work of breast cancer patients: results from the OPTISOINS01 French prospective study. BMJ Open. 2018;8(5):e020276. doi: 10.1136/bmjopen-2017-020276.

6. Majou D, Mekarnia Y, Martin B, Rouzier R, Hequet D. [Episode-based bundled payment model: evaluation of medical costs for early operable breast cancer]. Bull Cancer. 2021;108(12):1091-100. doi: 10.1016/j.bulcan.2021.07.006.

7. Ferrier C, Thebaut C, Levy P, Baffert S, Asselain B, Rouzier R, et al. Absenteeism and indirect costs during the year following the diagnosis of an operable breast cancer: A prospective multicentric cohort study. J Gynecol Obstet Hum Reprod. 2021;50(6):101871. doi: 10.1016/j.jogoh.2020.101871.

8. Lerebours F, Héquet D, Baffert S, Hoang H, Brédart A, Asselain B, et al. Optisoins01: Optimizing the patient-breast cancer care pathway; An observational multicentric prospective study (abstract). Cancer Res. 2016;76(4_Supplement). doi: <https://doi.org/10.1158/1538-7445.SABCS15-OT2-04-01>.

9. Agency for Information on Hospital Care (Atih). MCO and HAD tariffs [Public administrative institution]. 2022 [Available from: <https://www.atih.sante.fr/tarifs-mco-et-had>.

**
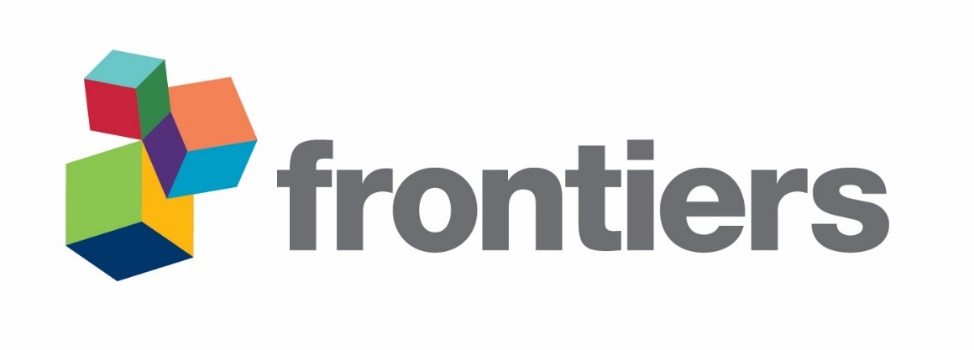
**
