# Supplementary material for: Genomic signature to guide adjuvant chemotherapy treatment decisions for early breast cancer patients in France: a cost-effectiveness analysis
Source: Front Oncol. 2023 Jun 23;13:1191943. doi: 10.3389/fonc.2023.1191943 (PMC10327821; doi:10.3389/fonc.2023.1191943)
Supplement: Supplementary Table 1 — Average number of CT cures in each population. [file Table_1.docx]

Supplementary Table

Supplementary Table 1. Average number of CT cures in each population

| Population | Average number of CT cycles | Min number of CT cycles | Max number of CT cycles |
| --- | --- | --- | --- |
| N0 < 50 with CT | 5.5 | 4 | 6 |
| N0 ≥ 50 with CT | 5.4 | 4 | 12 |
| N1 ≥ 50 with CT | 6.0 | 1 | 12 |
| **Overall pop. with CT** | **5.6** | **1** | **12** |
