# Supplementary material for: Genomic signature to guide adjuvant chemotherapy treatment decisions for early breast cancer patients in France: a cost-effectiveness analysis
Source: Front Oncol. 2023 Jun 23;13:1191943. doi: 10.3389/fonc.2023.1191943 (PMC10327821; doi:10.3389/fonc.2023.1191943)
Supplement: Supplementary Table 2 — Model parameters with DSA ranges and PSA distributions [file Table_2.docx]

Supplementary Table

# Sensitivity analyses parameters

The table below provides values and distribution of the parameters used for the DSA and PSA.

Supplementary Table 2. Model parameters with DSA ranges and PSA distributions

| Parameters | Base case value | Low value | High value | PSA distribution (parameters) | References |
| --- | --- | --- | --- | --- | --- |
|  |  |  |  |  |  |
| **Model settings** |  |  |  |  |  |
| Starting age (years) |  |  |  |  |  |
| N0 and age < 50 years^1^ | 43 | 31 | 49 | *Gamma (sd: 4.43)* | Lower bound 95%CI; 49 years |
| N0 and age ≥ 50 years^1^ | 64 | 50 | 87 | *Gamma (sd: 9.21)* | 50 years; Upper bound 95%CI |
| N1 and age ≥ 50 years^1^ | 61 | 50 | 79 | *Gamma (sd: 7.16)* | 50 years; Upper bound 95%CI |
| Time horizon^2^ | 57 | 10 | 20 | NA |  |
| Discount rate |  |  |  |  |  |
| costs <30y | 2.50% (1) | 1.50% | 5.0% | NA | (1) |
| costs >30y | 1.50% (1) | 0.0% | 2.50% | NA |  |
| outcomes <30y | 2.50% (1) | 1.50% | 5.0% | NA |  |
| outcomes >30y | 1.50% (1) | 0.0% | 2.50% | NA |  |
| Patients’ distribution |  |  |  |  |  |
| N0 <50 years | 41.2% (2, 3) | 53.0% | 29.5% | *Dirichlet (α_1_: 26, β_1_: 37)* | Complement to 100% |
| N0 ≥50 years | 16.7% (2, 3) | 13.3% | 20.0% | *Dirichlet (α_2_: 77, β_2_: 387)* | +/- 20% |
| N1 ≥50 years | 42.1% (2) | 33.7% | 50.5% | *Dirichlet (α_3_: 54, β_3_: 0)* | +/- 20% |
| **Clinical data** |  |  |  |  |  |
| RS distribution N0 <50y^3^ |  |  |  |  |  |
| RS 0-15 | 33.7% (3, 4) | 46.9% | 20.4% | *Dirichlet (α_1_: 674, β_1_: 1329)* | Complement to 100% |
| RS 16-25 | 39.3% (3, 4) | 31.4% | 47.1% | *Dirichlet (α_2_: 787, β_2_: 542)* | +/- 20% |
| RS 26-100 | 27.1% (3, 4) | 21.6% | 32.5% | *Dirichlet (α_3_: 542, β_3_: 0)* | +/- 20% |
| RS distribution N0 ≥50y^3^ |  |  |  |  |  |
| RS 0-25 | 71.8% (3, 4) | 77.5% | 66.2% | *Dirichlet (α_1_: 581, β_1_: 228)* | Complement to 100% |
| RS 26-100 | 28.2% (3, 4) | 22.5% | 33.8% | *Dirichlet (α_2_: 228, β_2_: 0)* | +/- 20% |
| RS distribution N1 ≥50y^3^ |  |  |  |  |  |
| RS 0-25 | 82.9% (5) | 86.3% | 79.5% | *Dirichlet (α_1_: 3353, β*_1_*: 692)* | Complement to 100% |
| RS 26-100 | 17.1% (5) | 13.7% | 20.5% | *Dirichlet (α_2_: 692, β*_2_*: 0)* | +/- 20% |
| CT probability (ODx) - N0 <50y |  |  |  |  |  |
| RS 0-15 | 0.0% (3) | 0.0% | 5.0% | Beta (α: 3, β: 334) | Arbitrary (0%, 5%) |
| RS 16-25 | 31.1% (3) | 24.9% | 37.3% | Beta (α: 122, β: 271) | +/- 20% |
| RS 26-100 | 100.0% (3) | 80.0% | 100.0% | Beta (α: 268, β: 3) | +/- 20% |
| CT probability (ODx) - N0 ≥50y |  |  |  |  |  |
| RS 0-25 | 0.0% (3) | 0.0% | 5.0% | Beta (α: 7, β: 711) | Arbitrary (0%, 5%) |
| RS 26-100 | 100.0% (3) | 80.0% | 100.0% | Beta (α: 279, β: 3) | +/- 20% |
| CT probability (ODx) - N1 ≥50y |  |  |  |  |  |
| RS 0-25 | 0.0% | 0.0% | 5.0% | Beta (α: 8, β: 821) | Arbitrary (0%, 5%) |
| RS 26-100 | 100.0% | 80.0% | 100.0% | Beta (α: 169, β: 2) | +/- 20% |
| CT probability (SoC) - N0 <50y | 60.8% (2, 6) | 48.7% | 73.0% | Beta (α: 37, β: 24) | +/- 20% |
| CT probability (SoC) - N0 ≥50y | 29.4% (2, 6) | 23.5% | 35.3% | Beta (α: 68, β: 162) | +/- 20% |
| CT probability (SoC) - N1 ≥50y | 77.6% (2, 6) | 62.1% | 93.2% | Beta (α: 21, β: 6) | +/- 20% |
| DRFS (ODx) – ET – N0 <50y |  |  |  |  |  |
| RS 0-15 | 97.0% (3, 4) | 94.1% | 99.2% | Beta (α: 143, β: 4) | 95% CI (Beta) |
| RS 16-25 | 82.7% (3, 4) | 76.6% | 87.6% | Beta (α: 150, β: 32) | 95% CI (Beta) |
| RS 26-100 | 71.1% (3, 4) | 65.0% | 76.7% | Beta (α: 162, β: 66) | 95% CI (Beta) |
| DRFS (ODx) – ET – N0 ≥50y |  |  |  |  |  |
| RS 0-25 | 90.4% (3, 4) | 88.4% | 92.3% | Beta (α: 776, β: 82) | 95% CI (Beta) |
| RS 26-100 | 70.2% (3, 4) | 67.3% | 73.0% | Beta (α: 702, β: 298) | 95% CI (Beta) |
| DRFS (ODx) – ET – N1 ≥50y |  |  |  |  |  |
| RS 0-25 | 91.2% (5) | 89.8% | 92.5% | Beta (α: 1609, β: 155) | 95% CI (Beta) |
| RS 26-100 | 62.0% (7) | 38.4% | 79.7% | Beta (α: 12, β: 8) | 95% CI (Beta) |
| DRFS (ODx) – CT – N0 <50y |  |  |  |  |  |
| RS 0-15 | 97.8% (3, 4) | 96.0% | 99.0% | Beta (α: 354, β: 8) | 95% CI (Beta) |
| RS 16-25 | 91.7% (3, 4) | 87.2% | 95.1% | Beta (α: 174, β: 16) | 95% CI (Beta) |
| RS 26-100 | 83.3% (3, 4) | 78.2% | 87.9% | Beta (α: 190, β: 38) | 95% CI (Beta) |
| DRFS (ODx) – CT – N0 ≥50y |  |  |  |  |  |
| RS 0-25 | 90.8% (3, 4) | 92.5% | 96.5% | Beta (α: 444, β: 25) | 95% CI (Beta) |
| RS 26-100 | 78.3% (3, 4) | 74.7% | 81.6% | Beta (α: 424, β: 118) | 95% CI (Beta) |
| DRFS (ODx) – CT – N1 ≥50y |  |  |  |  |  |
| RS 0-25 | 90.6% (5) | 89.2% | 91.9% | Beta (α: 1622, β: 169) | 95% CI (Beta) |
| RS 26-100 | 75.4% (7) | 77.6% | 82.5% | Beta (α: 801, β: 199) | 95% CI (Beta) |
| Reduction in DR rate in 11-15y - CT | 0.0% (Assumption) | 0.0% | 20.0% | NA | Arbitrary (0%, 20%) |
| Reduction in DR rate in 16y+ - CT | 0.0% (Assumption) | 0.0% | 20.0% | NA | Arbitrary (0%, 20%) |
| Reduction in DR rate in 11-15y - ET | 0.0% (8) | 0.0% | 20.0% | NA | Arbitrary (0%, 20%) |
| Reduction in DR rate in 16y+ - ET | 0.0% (8) | 0.0% | 20.0% | NA | Arbitrary (0%, 20%) |
| **Long-term AEs of CT** |  |  |  |  |  |
| Probability of AML | 0.62% (9) | 0.2% | 1.4% | Beta (α: 643, β: 4.0) | 95% CI |
| HR for CHF with CT | 1.61 (10) | 1.088 | 2.298 | *Log-normal (sd: 0.31)* | 95% CI |
| **Mortality** |  |  |  |  |  |
| Median OS in DR (months) | 63.9 (11) | 52.4 | 71 | *Log-normal (sd: 6.325)* | 95% CI |
| 5-year AML death probability | 76.0% (12) | 74.0% | 77.0% | Beta (α: 801, β: 199) | 95% CI |
| (2, 3)**Utilities** |  |  |  |  |  |
| Utility adjustment factor | 1.043 (13) | 0.939 | 1.148 | NA | +/- 10% |
| Utility in |  |  |  |  |  |
| Recurrence-free state | 0.860 (14) | 0.819 | 0.894 | Beta (α: 273, β: 45) | 95% CI adjusted to French utility |
| DR state | 0.715 (14) | 0.647 | 0.767 | Beta (α: 129, β: 52) | 95% CI adjusted to French utility |
| AML state | 0.271 (15) | 0.217 | 0.326 | Beta (α: 65, β: 174) | +/- 20% |
| CHF state | 0.551 (10) | 0.458 | 0.642 | Beta (α: 61, β: 50) | 95% CI from Beta estimated with SE |
| Utility decrement for CT | 0.040 (16) | 0.032 | 0.048 | Beta (α: 88, β: 2136) | +/- 20% |
| **Costs** |  |  |  |  |  |
| Test cost (ODx) | €1,849.50 | €1,480 | €2,219 | *Gamma (sd: 199.3)* | +/- 20% |
| Recurrence-free cost - Year 1 |  |  |  |  |  |
| N0 <50 years | €7,553 | €5,081 | €10,507 | *Gamma (sd: 1388.2)* | 95% CI |
| N0 ≥50 years | €5,945 | €2,629 | €10,591 | *Gamma (sd: 2052.2)* | 95% CI |
| N1 ≥50 years | €6,888 | €5,190 | €8,823 | *Gamma (sd: 928.4)* | 95% CI |
| Recurrence-free cost - Years 2-5 | €604 (17) | €483 | €725 | *Gamma (sd: 65.09)* | +/- 20% |
| Recurrence-free cost - Years 6+ | €604 (17) | €483 | €725 | *Gamma (sd: 65.09)* | +/- 20% |
| CDK 4/6 costs in DR state | €14,395 (18) | €11,516 | €17,274 | *Gamma (sd: 1551.2)* | +/- 20% |
| Disease management cost in DR state | €6,515.50 (17) | €5,212 | €7,819 | *Gamma (sd: 702.1)* | +/- 20% |
| AML one-off cost | €23,970 (19) | €19,176 | €28,764 | *Gamma (sd: 2583)* | +/- 20% |
| AML subsequent cost | €5,805.50 (17) | €4,644 | €6,967 | *Gamma (sd: 625.6)* | +/- 20% |
| CHF one-off cost | €1,888 (19) | €1,510 | €2,265 | *Gamma (sd: 203.4)* | +/- 20% |
| CHF subsequent cost | €1,148 (17) | €918 | €1,378 | *Gamma (sd: 123.7)* | +/- 20% |
| Terminal care | €4,606 (19) | €3,684.50 | €5,527 | *Gamma (sd: 496.3)* | +/- 20% |
| Transportation Costs - CT |  |  |  |  |  |
| N0 <50 years | €2,906 | €115 | €10,049 | *Gamma (sd: 2693.3)* | 95% CI |
| N0 ≥50 years | €1,738 | €2.40 | €8,526 | *Gamma (sd: 2394.4)* | 95% CI |
| N1 ≥50 years | €1,200 | €0.25 | €6,634 | *Gamma (sd: 1884.9)* | 95% CI |
| Transportation Costs - ET |  |  |  |  |  |
| N0 <50 years | €255 | €0.00 | €2,339 | *Gamma (sd: 732.1)* | 95% CI |
| N0 ≥50 years | €925 | €0.00 | €6,086 | *Gamma (sd: 1762.3)* | 95% CI |
| N1 ≥50 years | €337 | €8.54 | €1,244 | *Gamma (sd: 337.2)* | 95% CI |
| Sick Leave Related to CT |  |  |  |  |  |
| N0 <50 years | €6,316 | €236 | €22,029 | *Gamma (sd: 5913.5)* | 95% CI |
| N0 ≥50 years | €5,235 | €0.00 | €39,795 | *Gamma (sd: 11791.5)* | 95% CI |
| N1 ≥50 years | €10,609 | €1,336 | €29,275 | *Gamma (sd: 7407.6)* | 95% CI |
| Additional costs due to CT |  |  |  |  |  |
| N0 <50 years | €2,063 | €1.55 | €10,550 | *Gamma (sd: 2975.03)* | 95% CI |
| N0 ≥50 years | €4,479 | €56 | €18,023 | *Gamma (sd: 4949.2)* | 95% CI |
| N1 ≥50 years | €3,619 | €1,491 | €6,675 | *Gamma (sd: 1338.2)* | 95% CI |
| Costs of ET - N0 <50 years | €91 (18) | €73 | €109 | *Gamma (sd: 9.79)* | +/- 20% |
| Costs of ET - N0/N1 ≥50 years | €222.50 (18) | €178 | €267 | *Gamma (sd: 23.97)* | +/- 20% |

^1^ For simplification, menopause was assumed to occur at age 50; ^2^ Lifetime horizon was defined as sufficient for the patient cohort to reach 100 years of age. Therefore, the number of years in the model differed depending on the age at model entry and was 57 years for premenopausal women with N0 disease, 39 years for postmenopausal women with N1 disease, and 36 years for post-menopausal women with N0 disease; ^3^ For premenopausal women, three RS categories were considered: <16, 16–25, and >25; for postmenopausal N0 and N1 sub-populations, only two RS categories were considered: ≤ 25 and > 25. Abbreviations: CT, chemotherapy; N0, node-negative disease; N1, 1–3 invaded lymph nodes; ODx, Oncotype DX; RS, recurrence score; SoC, standard of care; ET, endocrine therapy; DRFS, disease recurrence-free survival; DR, Distant recurrence; AML, acute myeloid leukemia; HR, hazard ratio; CHF, chronic heart failure; OS, overall survival.

References

1. Haute Autorité de Santé (HAS) - French Health Authority. Choices in methods for economic evaluation – HAS. 2020 06/04/2020.

2. Dumas E, Laot L, Coussy F, Grandal Rejo B, Daoud E, Laas E, et al. The French Early Breast Cancer Cohort (FRESH): A Resource for Breast Cancer Research and Evaluations of Oncology Practices Based on the French National Healthcare System Database (SNDS). Cancers. 2022;14(11):2671. doi: 10.3390/cancers14112671.

3. Sparano JA, Gray RJ, Ravdin PM, Makower DF, Pritchard KI, Albain KS, et al. Clinical and Genomic Risk to Guide the Use of Adjuvant Therapy for Breast Cancer. N Engl J Med. 2019;380(25):2395-405. doi: 10.1056/NEJMoa1904819.

4. Sparano JA, Gray RJ, Makower DF, Pritchard KI, Albain KS, Hayes DF, et al. Adjuvant Chemotherapy Guided by a 21-Gene Expression Assay in Breast Cancer. N Engl J Med. 2018;379(2):111-21. doi: 10.1056/NEJMoa1804710.

5. Kalinsky K, Barlow WE, Gralow JR, Meric-Bernstam F, Albain KS, Hayes DF, et al. 21-Gene Assay to Inform Chemotherapy Benefit in Node-Positive Breast Cancer. N Engl J Med. 2021;385(25):2336-47. doi: 10.1056/NEJMoa2108873.

6. Curtit E, Vannetzel J-M, Darmon J-C, Roche S, Bourgeois H, Dewas S, et al. Results of PONDx, a prospective multicenter study of the Oncotype DX® breast cancer assay: Real-life utilization and decision impact in French clinical practice. The Breast. 2019;44:39-45. doi: 10.1016/j.breast.2018.12.015.

7. Sestak I, Buus R, Cuzick J, Dubsky P, Kronenwett R, Denkert C, et al. Comparison of the Performance of 6 Prognostic Signatures for Estrogen Receptor–Positive Breast Cancer: A Secondary Analysis of a Randomized Clinical Trial. JAMA Oncol. 2018;4(4):545. doi: 10.1001/jamaoncol.2017.5524.

8. Pan H, Gray R, Braybrooke J, Davies C, Taylor C, McGale P, et al. 20-Year Risks of Breast-Cancer Recurrence after Stopping Endocrine Therapy at 5 Years. N Engl J Med. 2017;377(19):1836-46. doi: 10.1056/NEJMoa1701830.

9. Moebus V, Jackisch C, Lueck H-J, du Bois A, Thomssen C, Kurbacher C, et al. Intense Dose-Dense Sequential Chemotherapy With Epirubicin, Paclitaxel, and Cyclophosphamide Compared With Conventionally Scheduled Chemotherapy in High-Risk Primary Breast Cancer: Mature Results of an AGO Phase III Study. JCO. 2010;28(17):2874-80. doi: 10.1200/JCO.2009.24.7643.

10. Hall PS, Smith A, Hulme C, Vargas-Palacios A, Makris A, Hughes-Davies L, et al. Value of Information Analysis of Multiparameter Tests for Chemotherapy in Early Breast Cancer: The OPTIMA Prelim Trial. Value in Health. 2017;20(10):1311-8. doi: 10.1016/j.jval.2017.04.021.

11. Hortobagyi GN, Stemmer SM, Burris HA, Yap Y-S, Sonke GS, Hart L, et al. Overall Survival with Ribociclib plus Letrozole in Advanced Breast Cancer. N Engl J Med. 2022;386(10):942-50. doi: 10.1056/NEJMoa2114663.

12. Mounier M, Meynadié M, Troussard X, Orazio S, Monnereau A, Cornet E. Survie des personnes atteintes de cance en France métropolitaine 1989-2018 - Leucémies aigües myléloïdes. 2020.

13. Szende A, Janssen B, Cabases J. Self-Reported Population Health: An International Perspective based on EQ-5D. Szende A, Janssen B, Cabases J, editors. Dordrecht (NL)2014. doi: 10.1007/978-94-007-7596-1.

14. Lidgren M, Wilking N, Jonsson B, Rehnberg C. Health related quality of life in different states of breast cancer. Qual Life Res. 2007;16(6):1073-81. doi: 10.1007/s11136-007-9202-8.

15. Younis T, Rayson D, Skedgel C. The cost-utility of adjuvant chemotherapy using docetaxel and cyclophosphamide compared with doxorubicin and cyclophosphamide in breast cancer. Curr Oncol. 2011;18(6):e288-96. doi: 10.3747/co.v18i6.810.

16. Campbell HE, Epstein D, Bloomfield D, Griffin S, Manca A, Yarnold J, et al. The cost-effectiveness of adjuvant chemotherapy for early breast cancer: A comparison of no chemotherapy and first, second, and third generation regimens for patients with differing prognoses. Eur J Cancer. 2011;47(17):2517-30. doi: 10.1016/j.ejca.2011.06.019.

17. French National Health Insurance (Ameli). Data pathologies 2020 [Available from: <https://data.ameli.fr/pages/data-pathologies/>.

18. French National Health Insurance (Ameli). French public database of medicines 2019 [Available from: <http://www.codage.ext.cnamts.fr/codif/bdm_it/>.

19. Agency for Information on Hospital Care (Atih). MCO and HAD tariffs [Public administrative institution]. 2022 [Available from: <https://www.atih.sante.fr/tarifs-mco-et-had>.

**
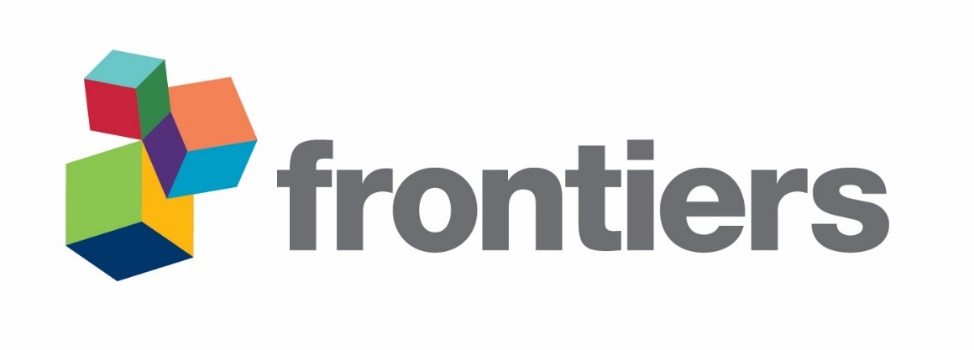
**
