# Supplementary figures and images for: Genomic signature to guide adjuvant chemotherapy treatment decisions for early breast cancer patients in France: a cost-effectiveness analysis
Source: Front Oncol. 2023 Jun 23;13:1191943. doi: 10.3389/fonc.2023.1191943 (PMC10327821; doi:10.3389/fonc.2023.1191943)

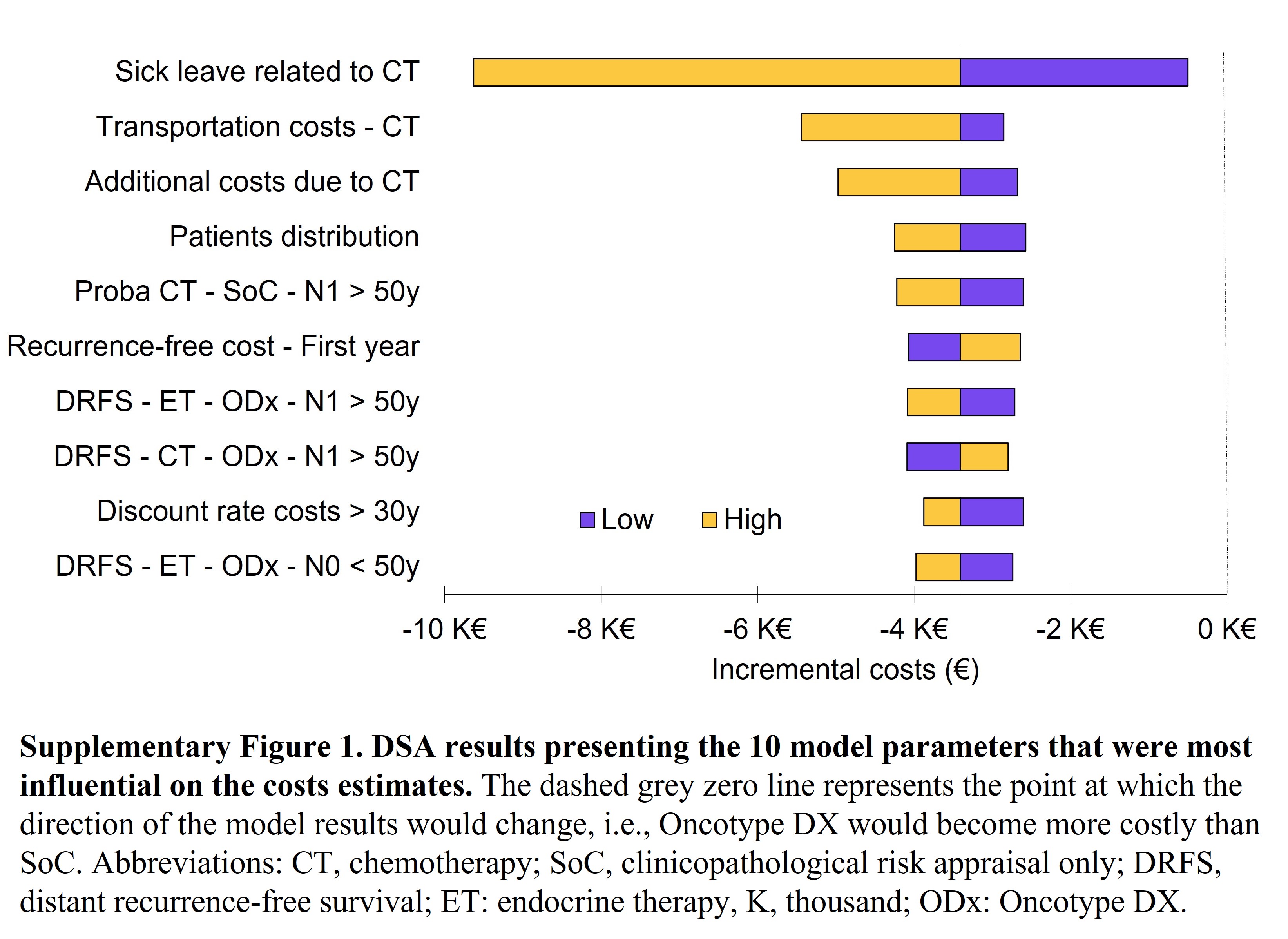

Supplement: Supplementary Figure 1 — DSA results presenting the 10 model parameters that were most influential on the costs estimates [file Image_1.jpeg]

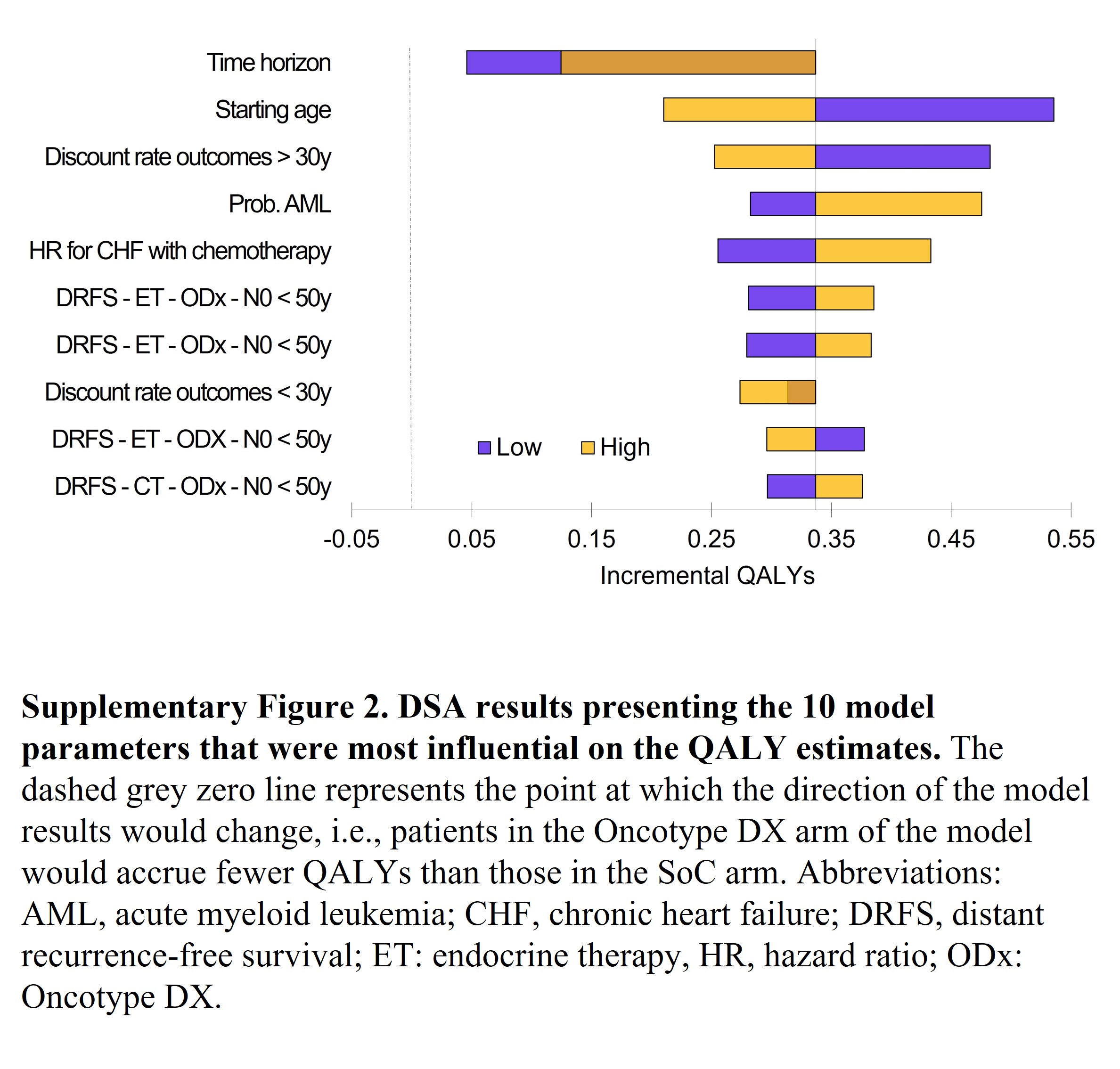

Supplement: Supplementary Figure 2 — DSA results presenting the 10 model parameters that were most influential on the QALY estimates. [file Image_2.jpeg]
